# Prevalence and determinants of infection risk in hospitalised adults: a multicentre cross-sectional study using a structured risk assessment tool

**DOI:** 10.3389/fpubh.2026.1872843

**Published:** 2026-07-23

**Authors:** Todo Bom Luís, Pragosa Ângela, Mata Ema, Costeira Cristina, Duarte Hugo, Ana Rita Mendes, Gonçalves Telma, Maria José Mendes, Cunha Helena, Parreira Ana, Maria dos Anjos Dixe, Marques Maria do Céu

**Affiliations:** 1 Comprehensive Health Research Centre (CHRC), University of Évora, Évora, Portugal; 2 ciTechCare - Centre for Innovative Care and Health Technology, Polytechnic of Leiria, Leiria, Portugal; 3 School of Health Sciences, Polytechnic University of Leiria, Leiria, Portugal; 4 Nursing Research Innovation and Development Centre of Lisbon (CIDNUR), School of Nursing, University of Lisbon, Lisbon, Portugal; 5 School of Nursing, University of Lisbon, Lisbon, Portugal; 6 The Health Sciences Research Unit: Nursing (UICISA: E), Coimbra, Portugal; 7 Local Health Unit of São José, Lisbon, Portugal; 8 Cascais Hospital, Cascais, Portugal; 9 Local Health Unit of the Region of Leiria, Leiria, Portugal

**Keywords:** healthcare-associated infections, hospitalised adults, infection prevention, infection risk, nursing assessment, patient safety, risk assessment

## Abstract

**Background:**

Healthcare-associated infections remain a major public health challenge, contributing to increased morbidity, mortality, length of hospital stays, and healthcare costs. Systematic assessment of infection risk is essential to support preventive strategies and improve patient safety however remains insufficiently structured in many clinical settings.

**Objective:**

To estimate the prevalence of infection risk among hospitalised adults and to identify intrinsic and extrinsic factors associated with high infection risk using a structured risk assessment instrument.

**Methods:**

A multicentre, observational, cross-sectional study was conducted in adult inpatient wards across multiple Local Health Units of the Portuguese National Health Service during 2025. Infection risk was assessed using the Risk Assessment for Infection (RAC) scale, which evaluates intrinsic and extrinsic risk factors. Risk stratification was performed using complementary approaches, including receiver operating characteristics (ROC) analysis and percentile-based classification. The study was conducted in accordance with ethical requirements.

**Results:**

A total of 1,092 hospitalised adults were included. Most participants were classified as having moderate (53.6%) or high (11.4%) infection risk according to percentile-based stratification. Higher risk levels were significantly associated with intrinsic factors such as older age, multimorbidity, impaired tissue integrity, and reduced mobility, as well as extrinsic factors including previous hospitalisation, intra- or inter-hospital transfer, prolonged length of stay, invasive procedures, and pharmacological therapy. ROC analysis identified a cut-off point with good discriminative performance, while percentile-based stratification enabled a more granular three-level risk classification. Patients hospitalised in medical wards had significantly higher risk scores compared with those in surgical wards.

**Conclusion:**

This study demonstrates a high prevalence of infection risk among hospitalised adults and confirms the multifactorial nature of infection risk in hospital settings. The RAC scale proved to be a useful tool for systematic risk stratification, with the three-level classification providing enhanced clinical applicability. Integrating structured infection risk assessment into routine care may support early identification of high-risk patients, enable targeted preventive interventions, and strengthen infection prevention and control programmes.

## 1 Introduction

Healthcare-associated infections (HAIs) remain a significant public health problem, associated with increased morbidity and mortality, prolonged hospital stays, and a significant rise in costs within the hospital setting (1). Despite the progress made in recent decades in terms of infection prevention and control strategies, the incidence of HAI remains high, particularly in hospital settings, where clinical complexity, exposure to invasive procedures and the coexistence of multiple risk factors increase the vulnerability of inpatients (2, 3).

The identification and monitoring of infection risk play a central role in infection prevention and control programmes, enabling support for clinical decision-making and the timely implementation of preventive interventions targeted at higher-risk groups. International recommendations emphasise the importance of epidemiological surveillance based on systematic and structured approaches, capable of generating comparable and useful data for the continuous improvement of the quality and safety of healthcare (World Health Organization, 2016; Organisation for Economic Co-operation and Development, 2019). However, in many clinical settings, the assessment of infection risk remains largely unsystematic, hindering the epidemiological characterisation of risk at institutional and national levels (4). Furthermore, recent literature reviews and leading international reports identify significant gaps in knowledge regarding the distribution of infection risk and its associated factors in hospital populations, highlighting the need for up-to-date, standardised and context-specific prevalence studies capable of capturing variability between institutions and regions and of supporting epidemiological surveillance and the planning of prevention strategies (5, 6).

Despite the growing body of scientific literature in this area, the available evidence has significant limitations. Most studies focus on the prevalence of established infections, with less emphasis on the prospective and systematic assessment of the risk of infection in hospitalised patients. Furthermore, many studies feature single-centre designs, methodological heterogeneity and a lack of validated tools for risk stratification, hindering the comparability of results and the generalisation of conclusions (7, 8). Recent reviews have also highlighted the scarcity of studies that simultaneously integrate intrinsic and extrinsic individual factors, as well as organisational variables, into the analysis of infection risk, highlighting the need for multicentre research using standardised methodologies and robust risk assessment tools (4, 5).

Assessing the prevalence of infection risk in hospitalised patients is a fundamental component of epidemiological surveillance, enabling the estimation of the magnitude of the problem, the identification of risk patterns, and the support of planning prevention strategies tailored to the clinical context. Globally, HAIs affect approximately 7% of hospitalised patients in more developed hospital settings and up to 15% in settings with greater structural limitations, reflecting inequalities in the organisation of care and the implementation of prevention measures (9). In Europe, recent data (6), covering the period 2022–2023, indicate that around 7.1% of patients admitted to acute care hospitals have at least one healthcare-associated infection, corresponding to approximately 4.3 million episodes annually, reflecting an increase compared to the data reported in the previous cycle of the European Centre for Disease Prevention and Control (2016–2017). In Portugal, the estimated prevalence is 7.8%, according to the results of the 2016–2017 National Prevalence Study (10). In this context, multicentre prevalence studies are particularly relevant, as they provide a comprehensive and comparable overview of risk distribution across different institutions and hospital populations, contributing to the setting of health priorities and the strengthening of infection prevention and control policies (11).

In this context, the aim of this study was to estimate the prevalence and stratification of infection risk among adults hospitalised in various Portuguese Local Health Units, as well as to determine the contribution of intrinsic and extrinsic factors to high infection risk among hospitalised adults. By providing robust epidemiological data on the risk profile in a hospital setting, this study aims to contribute to strengthening the scientific evidence in this area and to support the implementation of evidence-based infection prevention and control strategies tailored to different levels of risk.

## 2 Materials and methods

### 2.1 Study design

A multicentre observational study was conducted, following the recommendations of *the Strengthening the Reporting of Observational Studies in Epidemiology* (STROBE) guidelines for cross-sectional studies (12, 13).

### 2.2 Participants and setting

The study was initially planned as a multicentre study involving several Portuguese Local Health Units. Due to differences in ethical approval timelines, data collection was completed in three institutions, encompassing 15 inpatient wards, selected intentionally to represent different geographical regions of mainland Portugal (North, Centre and South), thereby covering distinct organisational and care contexts.

Data collection was carried out simultaneously across the participating institutions during a predefined period, ensuring the uniformity of collection procedures and the comparability of the prevalence estimates obtained. The study included adult inpatient wards in medical and surgical areas, covering various clinical specialities. Data were collected between December 2024 and July 2025.

### 2.3 Sample

The target population consisted of adults admitted to the wards included in the study. Individuals aged 18 years or older, with a length of stay of ≥72 h at the time of assessment and without a current diagnosis of infection, were considered eligible. This condition was verified by consulting the clinical record and obtaining confirmation from the healthcare professionals responsible for the patient, ensuring the absence of any documented infection at the time of hospital admission (14, 15). Individuals admitted on an outpatient basis, to accident and emergency departments, or to paediatric or obstetric units were excluded.

A population-based approach was adopted, including all adult inpatients who met the inclusion criteria in the participating departments at the time of data collection, provided they were able to understand and answer the questions asked, as assessed by the researcher at the time of data collection. Participants’ eligibility also depended on their cognitive ability to understand and answer the questions asked, which was assessed prior to data collection using the validated version of *the Mini-Mental State Examination* (MMSE) (16). Only patients who were conscious and oriented were included. This methodological choice is in line with the approach used in point prevalence studies in a hospital setting (6).

The final sample consisted of 1,092 hospitalised adults. The simultaneous application of this approach across multiple Local Health Units helped to reduce selection bias, thereby strengthening the methodological robustness and transferability of the results obtained in a real clinical setting. To minimise information bias, data collection was performed by trained researchers following a standardised protocol. Prior to data collection, all researchers received specific training regarding the application of the RAC scale and the procedures for data extraction and recording.

### 2.4 Instruments

Data collection was carried out via a structured interview, comprising two parts. The first part included sociodemographic and clinical variables, enabling the characterisation of the study population.

The second part incorporated the Risk of Infection Assessment Scale for Adult Patients (RAC), developed by Acela (2017), an instrument for identifying and stratifying the risk of infection in hospitalised adults. The scale assesses the risk of infection based on intrinsic factors (gender, age, smoking habits, alcohol consumption, nutritional status, comorbidities, presence of non-surgical injuries or wounds, and physical mobility) and extrinsic factors, namely previous hospitalisation, intra-or inter-hospital transfer, type of ward, length of stay, surgical intervention, exposure to invasive procedures, and pharmacological and/or non-pharmacological therapy. Each item is scored according to predefined criteria, allowing a total score to be obtained, where higher values reflect a greater accumulation of risk factors and, consequently, a higher probability of infection.

The cultural adaptation and content validation of the RAC scale into European Portuguese was carried out by Martins et al. (2025), following internationally recommended methodological procedures for the cross-cultural adaptation of instruments. The process included translation, synthesis, back-translation, face validity and content validity via a Delphi panel of experts, resulting in an overall content validity index of 95%, supporting the semantic, conceptual and practical adequacy of the Portuguese version.

The clinical variables collected corresponded to the intrinsic and extrinsic dimensions assessed by the RAC scale and were analysed both individually and within the structured infection risk assessment framework.

To achieve the objectives, this study assessed the discriminatory performance and defined cut-off points applicable to the clinical stratification of infection risk. To this end, two complementary statistical methods were used: analysis of the Receiver Operating Characteristic (ROC) curve and the determination of percentiles of the distribution of the scores obtained.

These 33 patients were not included in the prevalence sample and were used exclusively as an independent reference group for ROC analysis to assess the discriminatory performance of the RAC scale, compared with 1,092 participants without infection. This method allows the discriminatory power of the tool to be assessed, and the cut-off point to be identified that best distinguishes between individuals with and without the outcome under study, while maximising both sensitivity and specificity.

The 33 patients with infection were predominantly male (51.5%); over 60 years of age (87.9%), with only 12.1% aged between 41 and 59 years. 32 (97%) were admitted to a clinical/surgical ward and only 1 (3%) to intermediate care.

The optimal cut-off point identified was 18.5 points, with a sensitivity of 81.8% and a specificity of 74.7%, demonstrating good diagnostic performance of the scale.

However, ROC analysis inherently produces an essentially dichotomous classification (lower risk versus higher risk), which may limit intermediate clinical discrimination between individuals with distinct vulnerability profiles. Given that the risk of infection is a gradual and multifactorial phenomenon, it was additionally decided to use the percentile distribution of the scores observed in the sample, allowing for stratification into three clinically more informative risk levels, similar to that obtained in the original study (17, 18).

Thus, based on the 25th and 75th percentiles, the following categories were defined: low risk (4–14 points), moderate risk (15–20 points) and high risk (≥21 points). This approach allows for a more sensitive grading of risk, reducing the likelihood of classifying participants with intermediate risk into the low-risk group and facilitating the definition of preventive interventions proportionate to the identified level of vulnerability.

Thus, the combined use of ROC analysis and percentiles allowed for a balance between statistical rigour and clinical utility, while ensuring both the discriminatory power and practical applicability of the RAC Scale in the hospital setting.

Data collection was conducted by a research team comprising nurses from the participating institutions. All researchers involved received specific prior training on the study objectives, the procedures for applying the RAC Scale, the assessment criteria, and the completion of the instruments. The application of the scale was based on an interview with the in-patient, supplemented by direct observation and consultation of the clinical information available in the medical record at the time of assessment, ensuring the uniformity and standardisation of the data collection process.

### 2.5 Data analysis

The analytical strategy comprised four sequential stages: descriptive analysis of participant characteristics, bivariate analysis of associations between risk factors and infection risk categories, ROC curve analysis to evaluate the discriminatory performance of the RAC scale, and multivariable logistic regression to identify independent predictors of high infection risk.

The data were analysed using IBM Statistical Package for the Social Sciences (SPSS) Statistics, version 31.0.0.0 (IBM Corp., Armonk, NY, USA). A descriptive analysis of the variables was performed, using absolute and relative frequencies for categorical variables and measures of central tendency and dispersion for continuous variables, according to their distribution.

The prevalence of the risk of infection was estimated with 95% confidence intervals. To identify factors associated with the risk of infection, inferential analyses were performed using statistical tests appropriate to the type of variables and the distribution of the data. Where relevant, multivariate models were constructed to control for potential confounding factors. The level of statistical significance was set at 0.05.

To determine the cut-off points, Receiver Operating Characteristic (ROC) curve analysis was used, along with the determination of percentiles of the distribution of the scores obtained. Binary logistic regression was used to identify independent predictors of high infection risk, according to RAC risk stratification.

### 2.6 Ethical considerations

The study was conducted in accordance with the ethical principles of the Declaration of Helsinki and with applicable national legislation on the protection of personal data. The research protocol received approval from the Ethics Committees of the participating institutions, with data collection taking place between December 2024 and July 2025. All participants were fully informed about the study’s objectives and procedures and provided free, informed consent. Confidentiality, anonymity and data security were fully ensured throughout the entire research process.

## 3 Results

### 3.1 Sample characteristics

The sample consisted of 1,092 adult inpatients recruited from various Portuguese public hospitals. There was a slight predominance of males (52.5%), with 47.5% being female. In terms of age, the majority of participants were aged 60 or over (66.9%), followed by the 41–59 age group (20.8%) and the 18–40 age group (12.3%), indicating a predominantly older hospitalised population.

As for marital status, 38.1% were married or in a civil partnership, 28.1% were separated/divorced, 19.2% were widowed and 14.6% were single. With regard to educational qualifications, primary education was the most common (50.9%), followed by secondary education (23.4%), higher education—bachelor’s degree (15.8%), no formal schooling (7.3%) and postgraduate education (2.6%).

In terms of household composition, 44.1% lived with one person, 26.1% with two to three people, 21.9% lived alone and 7.9% with four or more people. As regards employment status, the majority were retired (59.3%), followed by those in employment (33.2%), the unemployed (6.7%) and students (0.7%).

As for region of residence, the majority lived in the Central Region (68.5%), followed by the Algarve (17.7%), Lisbon and the Tagus Valley (13.6%) and the North (0.3%).

In the care context, 92.7% were hospitalised in medical and/or surgical wards, while 7.3% were admitted to intermediate care units.

The descriptive analysis of the risk factors assessed by the Infection Risk Assessment Scale (RAC) revealed a high frequency of clinical conditions and healthcare exposures potentially associated with the development of infection in the study population.

Regarding intrinsic factors, it was found that 66.9% of participants were aged 60 years or older, reflecting a predominantly older adults hospitalised population. With regard to comorbidities, 53.8% had three or more concurrent chronic conditions. With regard to nutritional status, 13.3% were underweight and 34.0% were overweight. Non-surgical injuries or wounds were also observed in 27.4% of participants, of whom 7.7% had contaminated wounds. Regarding physical mobility, 23.2% required assistance or mobility aids and 11.4% were bedridden, indicating significant functional limitations in a considerable proportion of the sample.

Regarding extrinsic factors, 21.3% of participants had a documented history of hospitalisation within the preceding month. Furthermore, 68.0% had undergone intra or inter-hospital transfers prior to assessment, with 56.4% originating from critical care settings, including emergency departments, intensive care units, and recovery units. At the time of data collection, 44.2% of participants had been hospitalised for more than 7 days, while 18.8% had a length of stay of 16 days or longer.

Exposure to invasive procedures was frequent, occurring in 92.9% of the sample. Of these, 66.1% were low-complexity procedures, 11.0% were medium-complexity procedures and 15.8% were high-complexity procedures. With regard to pharmacological therapy, 57.4% were receiving antibiotics, antifungals or glucocorticoids, while 3.8% were receiving immunosuppressants, antineoplastic therapy or radiotherapy. Finally, 53.6% of participants had undergone surgical intervention during their hospital stay or in the preceding 12 months.

Overall, these results demonstrate a hospitalised population characterised by high clinical complexity, multimorbidity, functional dependence and significant exposure to invasive medical interventions, creating a context conducive to an increased risk of infection (Table 1).

**Table 1:** Distribution of responses regarding indicators of intrinsic and extrinsic factors for infection risk according to the RAC Scale (*n* = 1,092).

	Risk factor domain	Risk factor and category	No	%
Intrinsic factors	Gender	Female	519	47.5
		Male	573	52.5
	Age (years)	18–40	134	12.3
		41–59	227	20.8
		≥ 60	731	66.9
	Smoking habits	Non-smoker	742	67.9
		Former smoker	185	16.9
		Active/passive smoker	165	15.1
	Alcohol consumption	None or rarely	712	65.2
		Moderate	343	31.4
		High	37	3.4
	Nutritional classification (BMI)	Standard weight	576	52.7
		Underweight	145	13.3
		Overweight	371	34.0
	Comorbidities	None	275	25.2
		Up to 2 comorbidities	155	14.2
		≥ 3 comorbidities	588	53.8
		Immune system / oncology / transplantation	74	6.8
	Non-surgical injury or wound	No	793	72.6
		Clean	215	19.7
		Contaminated	84	7.7
	Mobility	Self-employed	715	65.5
		With assistance/aids	253	23.2
		Bedridden	124	11.4
Total intrinsic factors (mean and standard deviation)			7.9 ± 2.5	
Extrinsic factors	Previous hospitalisation (last month)	No	859	78.7
		Yes	233	21.3
	Intra/inter-hospital transfer	None	349	32.0
		Semi-critical care units	109	10.0
		Critical areas	616	56.4
		Other institution/home care	18	1.6
	Length of stay	1–7 days	610	55.9
		8–15 days	277	25.4
		≥ 16 days	205	18.8
	Invasive procedures	None	77	7.1
		Low complexity	722	66.1
		Medium complexity	120	11.0
		High complexity	173	15.8
	Pharmacological treatment	None	128	11.7
		Antacids/NSAIDs	295	27.0
		Antibiotics/antifungals/glucocorticoids	627	57.4
		Immunosuppressants/antineoplastic agents/radiotherapy	42	3.8
	Surgical intervention	No	507	46.4
		Clean surgery	354	32.4
		Clean-contaminated surgery	231	21.2
Total extrinsic factors (mean and standard deviation)			7.8 ± 2.3	
Total scale			15.7 ± 4.0	

The ward is associated with the risk of infection, as can be seen from the data in Table 2, with higher mean values in the intrinsic and extrinsic dimensions and in the total score among participants admitted to medical wards, compared with those in surgical wards (*p* < 0.01).

**Table 2:** Comparison of RAC scale scores by clinical department (*n* = 1,092).

Dimensions of the RAC scale	Medical area (Mean ± SD)	Surgical area (Mean ± SD)	*p*-value
Intrinsic factors	8.49 ± 2.42	7.86 ± 2.62	< 0.001
Extrinsic factors	8.19 ± 1.96	7.66 ± 2.49	0.002
Total RAC score	16.68 ± 3.55	15.52 ± 4.24	< 0.001

### 3.2 Prevalence of healthcare-associated infection risk

The prevalence of risk was assessed using two methods: a cut-off value (determined via the ROC curve) and the determination of percentiles.

According to the cut-off value of 18.5 determined via the ROC curve, it was found that 74.7% (816) of patients had no risk or low risk, while 25.3% (277) had high risk.

According to the cut-off points determined using percentiles, it was found that the majority of the study population was concentrated in the moderate-risk category, followed by the low-risk and high-risk groups. Overall, these results reflect a hospitalised population characterised by significant clinical complexity and exposure to multiple intrinsic and extrinsic factors associated with increased the risk. The distribution of risk levels is presented in Table 3.

**Table 3:** Distribution of infection risk levels according to the percentiles (*n* = 1,092).

RAC risk level	No.	%
Low risk	383	35.1
Moderate risk	585	53.6
High risk	124	11.4
Total	1,092	100.0

The comparison between the two stratification methods revealed statistically significant differences (χ^2^ = 496.308; *p* < 0.001), highlighting a discrepancy in the classification of risk levels between the dichotomous approach based on the ROC curve and stratification by percentiles.

#### 3.2.1 Independent predictors of high risk: binary logistic regression

Separate models were constructed for intrinsic and extrinsic factors in order to reduce analytical complexity and minimise potential effects of multicollinearity between variables. The most relevant data are shown in Table 4.

**Table 4:** Application of the chi-square test between risk stratification using the two calculation methods.

Risk stratification	No risk/low risk	At risk	x^2^	*p*
Low risk	383	0	496,308	<0.001
Moderate risk	433	152		
High risk	0	124		

In the model relating to intrinsic factors, reduced mobility, the presence of multiple comorbidities and the existence of a non-surgical injury or wound showed the strongest associations. Participants who were bedridden had a significantly higher probability of being classified as high infection risk (OR = 36.53; 95% CI: 18.45–72.31; *p* < 0.001). The presence of severe comorbidities also revealed a strong association (OR = 84.58; 95% CI: 26.24–272.64; *p* < 0.001).

In the model of extrinsic factors, Independent associations were found between high infection risk and previous hospitalisation, prolonged hospitalisation, highly complex invasive procedures, and complex pharmacological therapy. A length of stay of 16 days or more significantly increased the probability of infection (OR = 7.66; 95% CI: 4.83–12.13; *p* < 0.001), while highly complex invasive procedures had an OR of 14.53 (95% CI: 2.65–79.82; *p* = 0.002). Complex pharmacological therapy showed the greatest effect magnitude in the extrinsic model (OR = 109.45; 95% CI: 9.97–1201.77; *p* < 0.001).

Overall, the results suggest that high infection risk results from the interaction between prior clinical vulnerability and the intensity of healthcare exposure during hospitalisation (Table 5).

**Table 5:** Independent predictors associated with classification as high infection risk.

Variable		Odds ratio (OR)	95% confidence interval	*p*-value
Intrinsic factors	Bedridden status	36.53	18.45–72.3	< 0.001
	Severe comorbidities	84.58	26.24–272.64	< 0.001
	Contaminated wound	25.94	11.83–56.8	< 0.001
Extrinsic factors	Previous hospitalisation	2.51	1.66–3.80	< 0.001
	Hospitalisation ≥ 16 days	7.66	4.83–12.13	< 0.001
	Complex invasive procedures	14.53	2.65–79.82	0.002
	Complex pharmacological therapy	109.45	9.97–1,201.77	< 0.001

## 4 Discussion

This study characterised the prevalence of infection risk in hospitalised adults and identified the main associated intrinsic and extrinsic factors, through the application of a structured risk assessment tool in multiple Portuguese Local Health Units. These results are significant, as HAIs continue to represent a priority patient safety issue, with an impact on morbidity, mortality, length of stay and healthcare costs (1, 3, 6, 8).

A key contribution of this study lies in the use of two complementary approaches to classifying infection risk: ROC analysis and stratification by percentiles. ROC analysis enabled the identification of a cut-off point with good discriminatory performance, proving particularly useful for distinguishing individuals with a higher probability of infection. However, this approach results in a dichotomous classification, which may limit the clinical discrimination of intermediate risk levels. Given that infection risk is a multifactorial phenomenon influenced by the simultaneous presence of intrinsic and extrinsic factors, stratification by percentiles proved more clinically appropriate, allowing for the definition of three risk levels—low, moderate and high—and a more sensitive grading of participants’ vulnerability, in line with the original structure of the RAC Scale and its cultural adaptation into European Portuguese (17, 18).

The high proportion of participants classified as moderate or high risk suggests that a significant proportion of the hospitalised population presents multiple factors associated with increased infection risk, even in the absence of documented infection. This result is consistent with European prevalence studies, which demonstrate that cumulative exposure to healthcare, associated with advanced age, multimorbidity, functional dependence, invasive procedures and prolonged hospital stays, contributes to a high-risk burden in a hospital setting (6, 7, 9, 19). In this context, the assessment of risk prevalence, regardless of any existing infection, constitutes a relevant indicator of clinical vulnerability and a useful tool for epidemiological surveillance, resource planning and the definition of preventive strategies (1, 3, 6, 20).

The findings suggest that infection risk is associated with the interaction between intrinsic and extrinsic factors. In the intrinsic domain, advanced age, multimorbidity, the presence of a non-surgical injury or wound, and reduced mobility stand out as relevant determinants. The association between ageing and an increased risk of infection is widely described in the literature, and is explained by changes in the immune response, frailty, and a higher burden of chronic disease (21–23). Similarly, multimorbidity is associated with greater clinical vulnerability, greater exposure to complex care interventions, and a higher probability of adverse outcomes (21, 24).

The presence of a non-surgical injury or wound, particularly when contaminated, highlights the role of tissue integrity as a fundamental barrier to infection prevention. This finding is consistent with the literature on wound infection, which recognises the breakdown of the skin barrier, microbial contamination and impaired healing as determining factors in the development of infection (25–28). Reduced mobility, in turn, emerges as a marker of frailty and functional dependence, frequently associated with longer hospital stays, greater exposure to invasive devices, increased risk of respiratory infections and a greater need for nursing care (23, 29).

In contrast, the absence of a significant association with variables such as gender, smoking habits, alcohol consumption or nutritional status suggests that these factors have less discriminatory power in the hospital setting, when compared with clinical and functional determinants more directly related to vulnerability to infection, such as multimorbidity, frailty, tissue integrity and reduced mobility (21, 23, 24, 30).

With respect to extrinsic factors, the results reinforce the role of cumulative healthcare exposure in determining the risk of infection. Previous hospitalisation, intra- or inter-hospital transfers, prolonged length of stay, invasive procedures and complex pharmacological therapy were associated with higher levels of risk. These findings are consistent with studies identifying repeated hospital exposure, invasive devices, broad-spectrum antibiotic therapy, and pharmacological modulation of the immune response as structural determinants of healthcare-associated infections (6, 9, 27, 28, 31).

The absence of a significant association with surgical intervention alone suggests that the risk of infection does not depend exclusively on the surgical procedure itself, but rather on the overall care context, including length of stay, use of invasive devices, antimicrobial prophylaxis, underlying clinical condition, and perioperative prevention practices. This interpretation is consistent with international guidelines and previous studies that highlight the multifactorial nature of infections in a hospital setting, including surgical site infections (28, 31, 32).

Comparison by clinical area showed that participants admitted to medical wards had higher levels of infection risk compared to those admitted to surgical wards. This result may reflect the higher concentration of older adults patients, those with multimorbidity, prolonged hospital stays and greater functional dependence in medical wards, translating into greater clinical complexity and higher cumulative exposure to risk (7, 9, 19, 24). These findings reinforce the need for differentiated prevention strategies, tailored to the dominant risk profile in each care setting (1, 3, 9).

From a clinical practice perspective, the results of this study support the integration of systematic infection risk assessment as a central component of infection prevention and control programmes. The use of structured tools, such as the RAC Scale, enables the early identification of individuals in situations of greater vulnerability and supports clinical decision-making based on infection risk (17, 18). The three-level stratification is particularly relevant for defining differentiated intervention protocols, allowing the intensity of preventive measures to be tailored to the identified risk profile (1, 3, 9).

Thus, individuals classified as low risk may benefit from universal preventive measures; those at moderate risk should be subject to enhanced monitoring and interventions targeting modifiable factors; and high-risk individuals should be prioritised for intensive surveillance and prevention strategies, including systematic review of the need for invasive devices, implementation of early mobilisation protocols, structured monitoring of skin integrity, and coordination with infection prevention and control teams (1, 3, 25, 26, 29, 32).

This approach enables the implementation of a risk-based strategy, promoting the efficient use of resources and the implementation of early, targeted preventive measures. The integration of structured tools into clinical practice supports a paradigm shift, moving from a reactive approach, centred on established infection, to a proactive approach based on early risk identification, in line with international recommendations for patient safety, epidemiological surveillance and the prevention of HAIs (1, 3, 9, 20).

Despite the robustness associated with the multicentre design and the sample size, this study has some limitations. The cross-sectional design does not allow for the establishment of causal relationships between the identified factors and infection risk, being limited to the identification of associations, as is inherent in cross-sectional observational studies (12, 13). Moreover, it does not permit the assessment of temporal changes in infection risk throughout hospitalisation, nor the evaluation of the predictive performance of the RAC scale regarding the future occurrence of infection. Furthermore, although nine Portuguese healthcare units were contacted, only three obtained favourable ethical approval in a timely manner, which limited geographical representativeness. Data collection in a real clinical setting was also influenced by organisational constraints, namely workload and staff turnover. In addition, the multivariable analyses should be interpreted with caution because the number of outcome events was relatively limited in relation to the number of predictors included in the models, which may have contributed to unstable estimates and wide confidence intervals for some variables.

Future longitudinal studies are warranted to evaluate temporal changes in infection risk during hospitalisation and to further assess the predictive validity of the RAC scale regarding subsequent infection occurrence.

Future studies should explore the relationship between risk stratification and the actual occurrence of infections, as well as assess the impact of the systematic implementation of risk-stratified protocols on the reduction of HAIs. These results reinforce the relevance of systematic risk stratification as a central tool in the prevention of HAIs, supporting the need to integrate predictive models into clinical practice and to conduct longitudinal evaluations of their impact on health outcomes (1, 3, 9, 17, 18).

## 5 Conclusion

This multicentre study revealed a high prevalence of infection risk among hospitalised adults, with a predominance of moderate and high-risk levels, reflecting the increasing clinical, functional and care-related complexity of the hospital setting. The consistent association between intrinsic factors—namely advanced age, multimorbidity, compromised tissue integrity and reduced mobility—and extrinsic factors, such as cumulative care exposure, invasive procedures and pharmacological therapy, supports the multifactorial nature of infection risk at the time of assessment.

The use of a structured risk assessment tool, such as the Risk of Infection Assessment Scale (RAC), has demonstrated considerable relevance for the systematic and integrated stratification of risk in clinical settings, facilitating the early identification of the most vulnerable individuals and supporting the prioritisation of preventive interventions. The integration of complementary risk classification approaches—namely receiver operating characteristic (ROC) curve analysis and percentile-based stratification—further enhances the tool’s clinical utility. The latter is particularly valuable, as it enables the classification of individuals into three distinct risk levels.

Consistent application of this approach may contribute to more targeted and risk-proportionate management, enhancing the effectiveness of infection prevention and control programmes and the implementation of patient safety strategies at the institutional level, in line with international recommendations for the prevention of healthcare-associated infections (HAIs).

Overall, these results support the need to routinely integrate structured infection risk assessment into healthcare quality and safety programmes, promoting a shift from a reactive approach to a proactive one based on risk anticipation. Future longitudinal studies should explore the relationship between risk stratification and the actual occurrence of infections, as well as assess the impact of the systematic implementation of intervention models differentiated by risk level on the reduction of healthcare-associated infections.

## Data Availability

The datasets generated and/or analysed during the current study are available from the corresponding author upon reasonable request, subject to approval by the relevant ethics committees and participating institutions, and in accordance with applicable data protection and confidentiality regulations.

## References

[1] World Health Organization. Global Report on Infection Prevention and Control. 1st ed. Geneva: World Health Organization (2022). p. 1.

[2] Magill SS Edwards JR Bamberg W Beldavs ZG Dumyati G Kainer MA . Multistate point-prevalence survey of health care–associated infections. N Engl J Med. (2014) 370:1198–208. doi: 10.1056/NEJMoa1306801, 24670166 PMC4648343

[3] World Health Organization. Guidelines on core Components of Infection Prevention and Control Programmes at the national and acute Health care Facility level. Geneva: WHO (2016). 27977095

[4] Mori N Hirai J Ohashi W Asai N Shibata Y Mikamo H. Derivation of clinical predictive factors (CHIEF) for first recurrent Clostridioides difficile infection. Am J Infect Control. (2024) 52:419–23. doi: 10.1016/j.ajic.2023.10.004, 37832921

[5] Khatrawi EM Prajjwal P Farhan M Inban P Gurha S Al-ezzi SMS . Evaluating the knowledge, attitudes, and practices of healthcare workers regarding high-risk nosocomial infections: a global cross-sectional study. Health Sci Rep. (2023) 6:e1559. doi: 10.1002/hsr2.1559, 37701355 PMC10494663

[6] European Centre for Disease Prevention and Control Point Prevalence Survey of Healthcare-Associated Infections and Antimicrobial use in European acute care Hospitals – 2022-2023 (2024) Available online at: https://www.ecdc.europa.eu/en/publications-data/PPS-HAI-AMR-acute-care-europe-2022-2023

[7] Cassini A Plachouras D Eckmanns T Abu Sin M Blank HP Ducomble T . Burden of six healthcare-associated infections on European population health: estimating incidence-based disability-adjusted life years through a population prevalence-based modelling study. PLoS Med. (2016) 13:e1002150. doi: 10.1371/journal.pmed.1002150, 27755545 PMC5068791

[8] Hammoud S Amer F Lohner S Kocsis B. Patient education on infection control: A systematic review. Am J Infect Control. (2020) 48:1506–15. doi: 10.1016/j.ajic.2020.05.039, 32512081

[9] Suetens C Latour K Kärki T Ricchizzi E Kinross P Moro ML . Prevalence of healthcare-associated infections, estimated incidence and composite antimicrobial resistance index in acute care hospitals and long-term care facilities: results from two European point prevalence surveys, 2016 to 2017. Euro Surveill. (2016) 23:1800516. doi: 10.2807/1560-7917.ES.2018.23.46.1800516, 30458912 PMC6247459

[10] Directorate-General for Health Study on the Prevalence of Healthcare-Associated Infections and the use of Antimicrobials in Portuguese Hospitals 2016–2017 (2018) Available online at: https://www.sns.gov.pt/wp-content/uploads/2017/12/DGS_PCIRA_V8.pdf

[11] Organisation for Economic Co-operation and Development, Policies EO on HS and Portugal Country Health Profile 2019. State of Health in the EU. (2019)

[12] Vandenbroucke JP von Elm E Altman DG Gøtzsche PC Mulrow CD Pocock SJ . Strengthening the reporting of observational studies in epidemiology (STROBE): explanation and elaboration. Epidemiology. (2007) 18:805–35. doi: 10.1097/EDE.0b013e3181577511, 18049195

[13] von Elm E Altman DG Egger M Pocock SJ Gøtzsche PC Vandenbroucke JP . The strengthening the reporting of observational studies in epidemiology (STROBE) statement: guidelines for reporting observational studies. J Clin Epidemiol. (2008) 61:344–9. doi: 10.1016/j.jclinepi.2007.11.008, 18313558

[14] World Health Organization Patient Safety Curriculum guide: Multi-Professional edition. Topic 9: Infection Prevention and Control (2011)

[15] Damani N. Manual of Infection Prevention and Control. Occup Med. (2012)

[16] Folstein MF Folstein SE McHugh PR. “Mini-mental state”: A practical method for grading the cognitive state of patients for the clinician. J Psychiatr Res. (1975) 12:189–98. 1202204 10.1016/0022-3956(75)90026-6

[17] Acelas ALR Infection risk assessment scale for hospitalised adults: development and validation (2017), e41505.

[18] Martins M Bom LT Paiva-Santos F Dixe M d A Costeira C. Cultural adaptation and content validation of the RAC (Rodríguez-Almeida-Cañon) infection risk assessment scale for adult patients into European Portuguese. Millenn J Educ Technol Health. (2025) 28:e41505. doi: 10.29352/mill0228.41505

[19] Casalini G Pagani C Giacomelli A Galimberti L Milazzo L Coen M . Impact of a bundle of interventions on quality-of-care indicators for *Staphylococcus aureus* bacteraemia: a single-Centre, quasi-experimental, before–after study. Antibiotics (Basel). (2024) 13:646. doi: 10.3390/antibiotics13070646, 39061328 PMC11273465

[20] OECD. Health at a Glance 2019: OECD Indicators. Paris: Organisation for Economic Co-operation and Development (2019).

[21] Lazzaro A De Grolamo G Filippi V Innocenti GP Santinelli L Ceccarelli G . The interplay between host defence, infection, and clinical status in septic patients: a narrative review. Int J Mol Sci. (2022) 23:803. doi: 10.3390/ijms23020803, 35054993 PMC8776148

[22] Chudasama YV Zaccardi F Gillies CL Razieh C Yates T Kloecker DE . Patterns of multimorbidity and risk of severe SARS-CoV-2 infection: an observational study in the U.K. BMC Infect Dis. (2021) 21:908. doi: 10.1186/s12879-021-06600-y, 34481456 PMC8418288

[23] Hessels AJ Kuo YH Ahmed N. Epidemiology and impact of healthcare-associated infections in trauma patients: a national data analysis. Surg Infect. (2020) 21:871–6. doi: 10.1089/sur.2019.294, 32216703 PMC7703252

[24] Leaper D Assadian O Edmiston CE. Approach to chronic wound infections. Br J Dermatol. (2015) 173:351–8. doi: 10.1111/bjd.13677, 25772951

[25] Swanson T Ousey K Haesler E Bjarnsholt T Carville K Idensohn P . IWII wound infection in clinical practice consensus document: 2022 update. J Wound Care. (2022) 31:S10–21. doi: 10.12968/jowc.2022.31.Sup12.S10, 36475844

[26] Chen Z Song T Li Y Luo L Li Z Zhao Q. Risk factors for pulmonary infection in long-term bedridden patients: a meta-analysis. Am J Transl Res. (2021) 13:11014–25. 34786040 PMC8581881

[27] Ding X Tang Q Xu Z Xu Y Zhang H Zheng D . Challenges and innovations in treating chronic and acute wound infections: from basic science to clinical practice. Burns Trauma. (2022) 10:tkac014. doi: 10.1093/burnst/tkac014, 35611318 PMC9123597

[28] Berríos-Torres SI Umscheid CA Bratzler DW Leas B Stone EC Kelz RR . Centers for Disease Control and Prevention guideline for the prevention of surgical site infection, 2017. JAMA Surg. (2017) 152:784–91. doi: 10.1001/jamasurg.2017.0904, 28467526

[29] Yang Y Che K Deng J Tang X Jing W He X . Assessing the impact of frailty on infection risk in older adults: prospective observational cohort study. JMIR Public Health Surveill. (2024) 10:e59762. doi: 10.2196/59762, 39412881 PMC11498063

[30] Wang C Jiang W Yang K Yu D Newn J Sarsenbayeva Z . Electronic monitoring systems for hand hygiene: systematic review of technology. J Med Internet Res. (2021) 23:e27880. doi: 10.2196/27880, 34821565 PMC8663600

[31] Kaiser P Wächter J Windbergs M. Therapy of infected wounds: overcoming clinical challenges by advanced drug delivery systems. Drug Deliv Transl Res. (2021) 11:1545–67. doi: 10.1007/s13346-021-00932-7, 33611768 PMC8236057

[32] Czerniak B Banaś W Budzyński J. Healthcare-associated infection and unfavourable outcomes during a one-year follow-up after discharge: a single-Centre study. Int J Occup Med Environ Health. (2025) 38:179–89. doi: 10.13075/ijomeh.1896.02473, 40247721 PMC12064349

